# Generation of Platelet Microparticles after Cryopreservation of Apheresis Platelet Concentrates Contributes to Hemostatic Activity

**DOI:** 10.4274/tjh.2016.0049

**Published:** 2017-03-01

**Authors:** İbrahim Eker, Soner Yılmaz, Rıza Aytaç Çetinkaya, Aysel Pekel, Aytekin Ünlü, Orhan Gürsel, Sebahattin Yılmaz, Ferit Avcu, Uğur Muşabak, Ahmet Pekoğlu, Zerrin Ertaş, Cengizhan Açıkel, Nazif Zeybek, Ahmet Emin Kürekçi, İsmail Yaşar Avcı

**Affiliations:** 1 University of Health Sciences Gülhane Faculty of Medicine, Division of Pediatric Hematology, Ankara, Turkey; 2 University of Health Sciences Gülhane Faculty of Medicine, Blood Training Center and Blood Bank, Ankara, Turkey; 3 University of Health Sciences Gülhane Faculty of Medicine, Haydarpaşa Sultan Abdülhamid Training and Research Hospital, Department of Infectious Disease, İstanbul, Turkey; 4 University of Health Sciences Gülhane Faculty of Medicine, Division of Immunology and Allergy, Ankara, Turkey; 5 University of Health Sciences Gülhane Faculty of Medicine, Department of General Surgery, Ankara, Turkey; 6 Memorial Hospital, Division of Hematology, Ankara, Turkey; 7 University of Health Sciences Gülhane Faculty of Medicine, Division of Hematology, Ankara, Turkey; 8 University of Health Sciences Gülhane Faculty of Medicine, Division of Biostatistics, Ankara, Turkey; 9 University of Health Sciences Gülhane Faculty of Medicine, Department of Infectious Disease and Clinical Microbiology, Ankara, Turkey; 10 Retired

**Keywords:** Platelet, cryopreservation, Microparticle generation, Hemostatic activity

## Abstract

**Objective:**

In the last decade, substantial evidence has accumulated about the use of cryopreserved platelet concentrates, especially in trauma. However, little reference has been made in these studies to the morphological and functional changes of platelets. Recently platelets have been shown to be activated by cryopreservation processes and to undergo procoagulant membrane changes resulting in the generation of platelet-derived microparticles (PMPs), platelet degranulation, and release of platelet-derived growth factors (PDGFs). We assessed the viabilities and the PMP and PDGF levels of cryopreserved platelets, and their relation with thrombin generation.

**Materials and Methods:**

Apheresis platelet concentrates (APCs) from 20 donors were stored for 1 day and cryopreserved with 6% dimethyl sulfoxide. Cryopreserved APCs were kept at -80 °C for 1 day. Thawed APCs (100 mL) were diluted with 20 mL of autologous plasma and specimens were analyzed for viabilities and PMPs by flow cytometry, for thrombin generation by calibrated automated thrombogram, and for PDGFs by enzyme-linked immunosorbent assay testing.

**Results:**

The mean PMP and PDGF levels in freeze-thawed APCs were significantly higher (2763±399.4/µL vs. 319.9±80.5/µL, p<0.001 and 550.9±73.6 pg/mL vs. 96.5±49 pg/mL, p<0.001, respectively), but the viability rates were significantly lower (68.2±13.7% vs. 94±7.5%, p<0.001) than those of fresh APCs. The mean endogenous thrombin potential (ETP) of freeze-thawed APCs was significantly higher than that of the fresh APCs (3406.1±430.4 nM.min vs. 2757.6±485.7 nM.min, p<0.001). Moreover, there was a significant positive poor correlation between ETP levels and PMP levels (r=0.192, p=0.014).

**Conclusion:**

Our results showed that, after cryopreservation, while levels of PMPs were increasing, significantly higher and earlier thrombin formation was occurring in the samples analyzed despite the significant decrease in viability. Considering the damage caused by the freezing process and the scarcity of evidence for their in vivo superiority, frozen platelets should be considered for use in austere environments, reserving fresh platelets for prophylactic use in blood banks.

## Abstract (tr)

**Amaç:**

Son on yıl içerisinde, dondurulup saklanan trombositlerin özellikle travma hastalarında kullanımı ile ilgili önemli bir bilgi birikimi oluşmuştur. Bununla birlikte bu çalışmalarda trombositlerin morfolojik ve fonksiyonel değişikliklerinden çok az bahsedilmektedir. Son zamanlarda dondurulup saklanan trombositlerin aktive olarak trombosit kaynaklı mikropartikül (TKM) oluşumu ve trombosit kaynaklı büyüme faktörü (TKBF) salınımı ile sonuçlanan prokoagulan membran değişiklikleri olduğu saptanmıştır. Çalışmamızda dondurulup saklanan trombositlerin canlılıkları, TKM ve TKBF düzeyleri incelenerek trombin olumuyla ilişkileri değerlendirildi.

**Gereç ve Yöntemler:**

Yirmi bağışçıdan alınan aferez trombosit süspansiyonları (ATS) bir gün bekletildikten sonra %6 dimetil sülfoksid ile dondurularak -80 °C’de bir gün saklandı. Trombositler eritildikten sonra 20 mL. otolog, plazma ile seyreltildi ve alınan örneklerden incelemeler yapıldı.

**Bulgular:**

Dondurulup çözülmüş ATS’lerdeki TKM seviyeleri tazeATS’lerdekinden anlamlı düzeyde daha yüksekti (2763±399,4/µL ve 319,9±80,5/µL; p<0,001). Dondurulup çözülmüş ATS’lerdeki PDGF seviyeleri de taze ATS’lerdekinden anlamlı düzeyde daha yüksekti (550,9±73,6 pg/mL ve 96,5±49 pg/mL; p<0,001). Bununla birlikte dondurulup çözülmüş ATS’lerin canlılıkları taze ATS’lere göre anlamlı düzeyde düşüktü (68,2±13,7% ve 94±7,5%, p<0,001). Dondurulup çözülmüş ATS’lerin ortalama endojen thrombin potensiyelleri (ETP) taze ATS’lerinkinden istatiksel olarak anlamlı düzeyde yüksekti (3406,1±430,4 nM.min ve 2757,6±485,7 nM.min, p<0,001). Ayrıca ETP ile TKM düzeyleri arasında istatiksel olarak anlamlı zayıf pozitif korelasyon mevcuttu (r=0,192, p=0,014).

**Sonuç:**

Çalışmamızdaki sonuçlar dondurulup çözüldükten sonra ATS’lerdeki trombositlerin canlılıklarında önemli düzeyde azalma olsa da, daha erken ve daha yüksek trombin oluşumunun gerçekleştiğini ve bunun da dondurma işlemi sonrası anlamlı düzeyde artan TKM’ler ile ilişkili olarak meydana geldiğini göstermektedir. Dondurma işlemi ile trombositlerde hasarlanmalar meydana gelmektedir ve dondurulmuş trombositlerin in vivo kullanımlarının taze trombositlere göre üstünlüğü ile ilgili bilimsel kanıtlar yetersizdir. Bu sebeple dondurulmuş trombosit süspansiyonlarının travma şartlarında kullanılmalarının ve taze trombosit süspansiyonlarının ise kan bankalarında, profilaktik kullanım amacıyla bulundurulmalarının daha uygun olacağı değerlendirilmiştir.

## INTRODUCTION

In blood banking practice, platelet concentrates prepared from apheresis devices or whole blood donations have a shelf life of up to 7 days. In order to overcome short shelf life-related logistic problems, Klein et al. investigated the use of previously cryopreserved platelets in a bleeding thrombocytopenic patient. Their study was carried out in the 1950s. Since then, numerous in vivo and in vitro studies have been published [1,2]. Dimethyl sulfoxide (DMSO) is the most common method for freezing platelets, but the removal of DMSO can be done before freezing or after thawing platelets. After thawing, platelet concentrate may be diluted with plasma, normal saline, or platelet additive solutions. These processes may yield an in vitro platelet recovery of 70%-80% [3].

While fresh liquid platelets have a short shelf life, frozen platelets can be stored for up to 3 years [4]. Autologous, allogenic, ABO, and human leukocyte antigen-compatible platelet transfusions are benefits of cryopreserved platelets in patients with refractoriness to prophylactic platelet transfusion. Natural disasters and large-scale military conflicts may cause austere environments in which cryopreserved platelets may serve as the sole platelet resources for the treatment of trauma-associated coagulopathy. Moreover, platelet leukocyte antigen 1 negative and type-specific platelet stores for human leukocyte antigen compatibility tests are among the numerous benefits [5].

Due to the ease of transportation and because no laboratory tests are required for their transfusion in emergency settings, various countries store cryopreserved platelets as part of their national contingency plans for natural disasters, terror attacks, and large-scale military conflicts. Extensive use of frozen platelets has been adopted by various armies, including in Australia [6] and Spain [7], which have published their experiences. However, scientific evidence for their efficacy is scarce. Besides the advantages of the transfusion of frozen platelets on hemostatic activity, there are deleterious effects that the freezing and thawing processes exert on platelet functions, which become manifest by in vitro tests.

A recently reported deleterious effect of platelet cryopreservation is the activation and procoagulant membrane changes of platelets, resulting in the generation of platelet-derived microparticles (PMPs). Platelet activation also leads to the degranulation and release of platelet-derived growth factors (PDGFs) [3,8]. Information on the contribution of the activation and procoagulant membrane changes of platelets after cryopreservation to the hemostatic activity and to the PDGF content of the platelet concentrates is scarce in the literature. As such, the purpose of our study was to explore whether and to what extent the activation and procoagulant changes of platelet membranes after cryopreservation affect the in vitro hemostatic activity and PDGF content of apheresis platelet concentrates (APCs).

## MATERIALS AND METHODS

In August 2013, University of Health Sciences Gülhane Faculty of Medicine Ethics Committee approval was received for the assessment of in vitro hemostatic activity of cryopreserved APCs. Platelet concentrates were obtained using the apheresis method (Trima, CaridianBCT, Inc., Lakewood, CO, USA) from 20 donors that met the national blood and blood products criteria for the donation of APCs. A total of 200 mL of APCs was collected in acid-citrate-dextrose (ACD, NIH, Formula A, Baxter Healthcare Corp., Deerfield, IL, USA) at a ratio of 1 volume of ACD to 10 volumes of blood. APCs were divided into two volume packs of 100 mL each. Before freezing, APCs were preserved in an automatic shaker (horizontal plane, at 20-24 °C) for 1 day.

### Freezing Process of Apheresis Platelet Concentrates

Due to the fact that plasma-reconstituted cryopreserved platelets are more procoagulant than those reconstituted in PAS-G or 0.9% NaCl [3], all of the APCs were collected/stored and diluted with autologous plasma rather than the additive solution used for the cryopreservation procedure. The methods used for freezing in our study were based on the previously published methods of Valeri et al. [9]. Plasma collected by apheresis from each donor (41 mL) and 9 mL of 27% DMSO were mixed in an empty blood bag located on a rigid ice pack. The resultant 50 mL mixture and 100 mL of APCs were collected in a 750 mL ethyl vinyl acetate freezing bag through a sterile hose combining device. The final DMSO concentration in the freezing bag was 6% and the bag was centrifuged at 22 °C and 1250 g for 10 min (Thermo Fisher Scientific RC12BP, Asheville, NC, USA). A platelet pellet of 20-25 mL was obtained after removal of the supernatant and the bag was put in a cardboard freezing box and stored at -80 °C [9].

### Thawing of Frozen Apheresis Platelet Concentrates

Cryopreserved platelets were stored for 24 h at -80 °C and then thawed by immersion in 37 °C water for 10 min. According to Valeri et al. [9], 50 mL of plasma is to be added after the thawing of 200-300 mL of cryopreserved APCs. However, in the current study, the volume of the APCs that underwent cryopreservation was 100 mL. Thus, we added 20 mL of freshly thawed plasma. Prior to testing, the thawed platelets were kept at room temperature for 30 min without agitation, as explained by Valeri et al. [9].

### In Vitro Measurements

All analyses were performed in the fresh state before freezing and after diluting the APCs in the post-thaw period. The fresh and frozen APCs were analyzed for the determination of platelet counts with a whole blood analyzer device (ABX Pentra XL80, HORIBA ABX SAS, Montpellier, France).

### Thrombin Generation Testing

Thrombin generation tests (TGTs) were performed with a calibrated automated thrombogram (CAT^®^, Thrombinoscope BV, Maastricht, the Netherlands) device that uses a slow-acting fluorogenic substrate instead of a chromogenic substrate for the latest TGTs. Thrombin generation closely correlates with platelet concentration. Thus, prior to testing, the platelet counts were normalized for the TGT. In TGTs, thrombin generation occurs in the presence of both phospholipid and tissue factor, which are present in either the platelet supernatant and/or the reagents. The platelet-rich plasma reagent (Thrombinoscope BV, the Netherlands) contains 1 pmol/L tissue factor and is used to assess the presence of phospholipid in the sample. The thrombin generation assays were performed 30 min after the thawing process.

A sample of 80 µL was collected from both dilution groups. Each sample was transferred to three different microtitrated plates (Immulon 2HB, Thermo Electron Corporation, Milford, MA, USA) that involved 20 µL of platelet-rich plasma reactant and 20 µL of thrombin calibrator. After the incubation of the mixture at 37 °C for 15 min, a sample of 20 µL was collected and added to 20 µL of Fluo-Buffer^®^ solution, and the reaction was monitored with a fluorometer. Using the Trombinoscope^®^ program, thrombogram curves, endogenous thrombin potentials (ETPs), and peak heights were measured. The area under the curve, which indicates the total amount of endogenous thrombin generated, was recorded as nmol/L x minute. The peak height, which indicates the highest thrombin value measured, was shown as nmol/L [10].

### Isolation and Quantitation of Microparticles

Flow cytometric analysis was used to quantify and characterize PMPs, which were identified by their size and the use of monoclonal antibodies (mAb) to determine the cellular origin. Analysis of PMPs was performed by adding 20 µL of CD41a fluorescein isothiocyanate (FITC; BD, USA) and CD62P phycoerythrin (PE; BD, USA) antibodies and 50 µL of sample to Trucount tubes (BD, USA). Tubes were incubated in the dark at room temperature for 20 min. After incubation, samples were suspended with 1 mL of phosphate buffered saline, which contained 1% paraformaldehyde. All samples were analyzed immediately with a FACSCanto II flow cytometer and FACSDiva software (Becton Dickinson, USA). The platelet microparticles express phosphatidylserine, which is detected by annexin V labeling [3]. During the process with the Annexin V Apoptosis Detection Kit (BioLegend, USA), 5 µL of annexin V and 10 µL of 7-AAD solutions were added over 100 µL of sample and incubated in the dark at room temperature for 15 min. Annexin V binding buffer (400 µL) was added and analyzed by flow cytometry.

**Figure 1:**
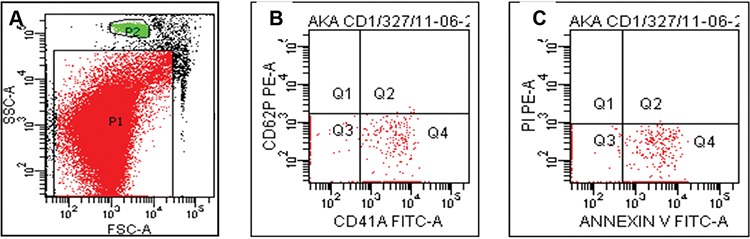
Isolation and quantitation of microparticles in freeze-thawed apheresis platelet concentrate samples. A) P1 is showing the gate of annexin-bound microparticles, which are selected in comparison with 1.0 µm latex beads (P2). B) Q1 is showing CD62P (+) and CD41a (-) platelet-derived microparticles, Q4 is showing CD62P (-) and CD41a (+) platelet-derived microparticles. C) Q4 is showing CD62P (-) and CD41a (+) and annexin V (+) platelet-derived microparticles.

Flow cytometric determination of PMPs was performed by using 1.0 µL beads (LB 8, Sigma, St. Louis, MO, USA). These beads were used to mark microparticle gates in order to confirm the PMP size. Forward scatter (FSC) and side scatter (SSC) were set to logarithmic gain for sample assessment. For the calculation of PMP absolute number, 20,000 event measurements were performed in Trucount tubes. Annexin V-positive, CD41a-positive, and CD62P-negative microparticles were defined as PMPs (Figure 1). The absolute number of PMPs per µL was calculated from the appropriate dot-plot values entered into the following formula [11]:

Number of events in the PMP region (P1) x Total number of beads per tube / Number of beads collected (P2) x Test volume (µL)

### Viability Evaluation Assays

**Figure 2:**
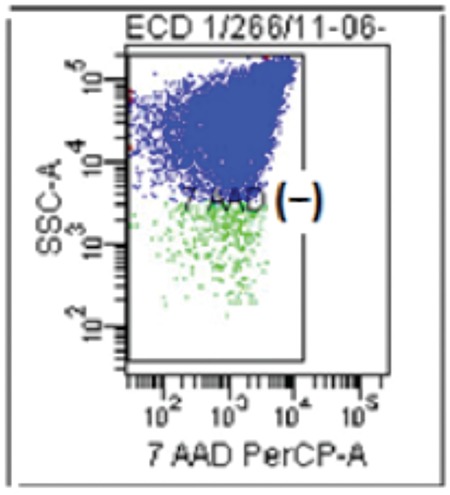
Viability evaluation assays showing the 7-AAD (-) unstained freeze-thawed platelets.

Assays were performed with 7-AAD (actinomycin D analog), which binds to DNA and was initially used in chromosome analysis, cell cycle studies, and the quantification of apoptosis. To date, 7-AAD staining followed by flow cytometry analysis is one of the most widely established assays for viability evaluation. 7-AAD has the ability to penetrate the cell membrane and complex; the DNA of dead cells, however, cannot be penetrated. Platelets do not contain a nucleus, but they are rich in mitochondria [12]. Cell death and injury often lead to release or exposure of intracellular molecules called damage-associated molecular patterns (DAMPs) or cell death-associated molecules. The mitochondrial DNA (mtDNA) can also function as a DAMP [13]. The mtDNA is released from dying or dead cells, with which 7-AAD has the ability to complex [14]. Cell viability was assessed by an assay using FITC-conjugated annexin V and 7-AAD. Briefly, samples were suspended in 100 µL of annexin binding buffer containing 5 µL of FITC-conjugated annexin V (1:5 dilution) and 10 µL of 7-AAD (100 µg/mL) and incubated at room temperature for 15 min. After the incubation period, 400 µL of annexin binding buffer was added. Samples were then immediately analyzed with a FACSCanto II flow cytometer and FACSDiva software (Becton Dickinson, USA) (Figure 2). In the total cell population analyzed, cells unstained and stained with 7-AAD were reported as a percentage of live and dead cells, respectively (Table 1).

### Platelet-Derived Growth Factors

An enzyme-linked immunosorbent assay (ELISA) test was performed to analyze PDGFs by using Human PDGF-BB ELISA kits (RayBiotech, Norcross, GA, USA). Absorbance of the ELISA plate was read and concentrations were assessed on an EL800x microplate reader [15].

### Statistical Analysis

Data were analyzed using computer software (IBM SPSS Statistics 22, licensed SPSS program of University of Health Sciences Gülhane Faculty of Medicine). Descriptive statistics were reported as frequencies and percentages for categorical variables and as mean ± standard deviation (SD) for continuous variables. As the one-sample Kolmogorov-Smirnov test showed that the variables were normally distributed, parametric analyses were performed. The Student t-test was used to compare continuous variables between groups. The Pearson correlation coefficient was calculated to evaluate the relationships between variables. Statistical significance was set at 0.05.

## RESULTS

**Figure 3:**
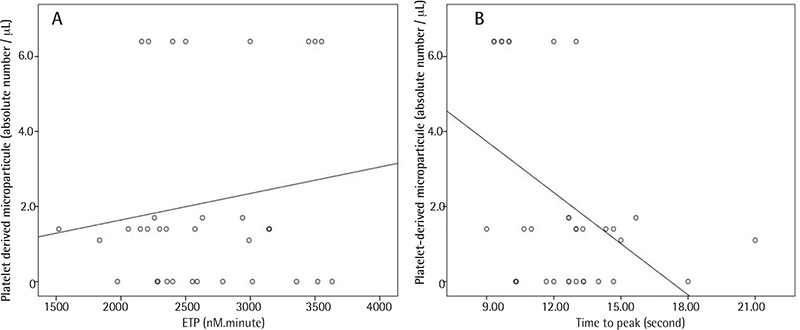
Correlation analyses related to platelet-derived microparticles. A) Scatterplot with linear fit graph of correlation analysis between platelet-derived microparticle levels and endogenous thrombin potential levels of freeze-thawed apheresis platelet concentrates. B) Scatterplot with linear fit graph of correlation analysis between platelet-derived microparticle levels and time to peak thrombin levels of freeze-thawed apheresis platelet concentrates.

There was no significant difference between the mean platelet counts of APCs before and after cryopreservation [(1195.2±153.5)x10^3^/µL and (1167±158.9)x10^3^/µL, respectively]. According to the TGT results, the mean ETP levels of freeze-thawed APCs were statistically significantly higher (3406.1±430.4 nM.min vs. 2757.6±485.7 nM.min, p<0.001), mean lag times were statistically significantly shorter (7.5±6.3 s vs. 9±2.2 s, p<0.001), and mean times to peak thrombin levels were statistically significantly shorter (12.6±5.4 s vs. 14.3±2.5 s, p<0.001) than those of the fresh APCs. According to the flow cytometric test results, the mean PMP levels of freeze-thawed APCs were statistically significantly higher (2763±399.4 absolute count/µL vs. 319.9±80.5 absolute count/µL, respectively; p<0.001), but the mean viability rate was statistically significantly lower (68.2±13.7% vs. 94±7.5%, respectively; p<0.001) than that of fresh APCs. According to ELISA test results, the mean PDGF levels of freeze-thawed APCs were statistically significantly higher (550.96±73.6 pg/mL vs. 96.4±49 pg/mL, respectively; p<0.001) than those of fresh APCs (Table 1). Moreover, there were statistically significantly positive poor correlations between the PMP levels and the ETP levels of freeze-thawed APCs (r=0.192, p=0.014) (Figure 3A). There were also statistically significant negative correlations between the PMP levels and time to peak thrombin levels of freeze-thawed APCs (r=-0.172, p=0.029) (Figure 3B). These results showed that, after cryopreservation, while levels of PMPs were increasing, significantly higher and earlier thrombin formation was occurring in the samples analyzed despite the significant decrease in viability.

## DISCUSSION

In this study, platelets were cryopreserved in 6% DMSO at -80 °C and reconstituted in plasma upon thawing. Results demonstrated that cryopreserved platelets generate high numbers of annexin V (phosphatidylserine)-expressing microparticles and PDGFs. Furthermore, our results suggest that cryopreservation of APCs increases their hemostatic activity via the PMP-related formation of significantly earlier and higher thrombin, despite the significant decrease in their viabilities.

It was demonstrated that phosphatidylserine-expressing PMPs support normal coagulation through the assembly of the FXa- and thrombin-generating coagulation enzyme complexes [16]. It has also been suggested that PMPs are up to 100-fold more procoagulant than platelets [17]. Our results, similar to those of Johnson et al. [3], confirmed the contribution of PMPs to the global coagulation potential of cryopreserved APCs.

TGTs have been used for identifying bleeding and hypercoagulability disorders in patients [18]. Our results suggest that cryopreserved platelets are hypercoagulable, as evidenced by a reduced lag time and time to peak and an increased thrombin generation potential (ETP) compared to the pre-freeze period. Moreover, there were statistically significantly positive correlations between the ETPs and PMPs, as well as statistically significantly negative correlations between PMP levels and time to peak thrombin. Thus, our results showed that, after cryopreservation, while levels of PMPs were increasing, significantly higher and earlier thrombin formation was occurring in the samples analyzed.

Besides the generation of PMPs, platelet activation via the cryopreservation process also leads to the release of granule contents within platelets. These granules are repositories for PDGFs and many coagulation factors [3,8,19]. After the freezing/thawing process, the levels of PDGFs in the APCs were 5.6-fold higher than those of the fresh APCs. Our results, similar to those of Ronci et al., also provide a rationale for using cryopreserved platelets in regenerative medicine [20]. Ronci et al. studied the release kinetics of PDGFs in homologous platelet-rich plasma, which was obtained from a platelet-apheresis procedure, and used it for the treatment of persistent ocular epithelial defects. To activate the platelets, they only used a cycle of freezing/thawing, without using a fibrin matrix as a support element or calcium chloride or thrombin for platelet activation. Similar to the results of our study, the levels of PDGF in the homologous platelet-rich plasma obtained from the platelet-apheresis procedure were 6.3-fold higher than the levels before the freezing/thawing process. All patients improved clinically during the follow-up period and the authors suggested that high levels of platelet counts were not required to treat corneal lesions when platelet-rich plasma was activated by a cycle of freezing/thawing.

While our study and that of Johnson et al. [3] suggest that cryopreserved platelets may have greater hemostatic potential than liquid-stored platelets, there are deleterious effects that the processes of freezing and thawing have on platelet functions, as demonstrated by in vitro tests. Frozen platelet adhesion is significantly decreased when compared to both fresh platelets and platelets stored for >5 days [21]. Recovery, survival, and other in vitro function markers, such as stimulus-response coupling, aggregation, granule release, and pH, are also impaired in frozen platelets [22,23,24]. It has also recently been reported that frozen and thawed platelets showed reduced surface expression of GPIIb and GPIbα and diminished aggregation response to agonists [25]. In a more recent study designed to evaluate the in vitro hemostatic efficacy of frozen versus fresh platelet transfusions by rotational thromboelastometry, a dual effect in frozen platelet transfusion was found: a hypercoagulable state (shortening of clotting time) and a more predominant impairment of frozen platelet functions when compared to fresh platelets (shorter maximum clot firmness/maximum clot elasticity and longer clot formation time) [26].

Despite these conflicting results, cryopreserved platelets have been used with great success in military operations since 2001, with more than 1000 units transfused to at least 333 patients [27]. Khuri et al. reported that the in vivo hemostatic functions of cryopreserved platelets in cardiopulmonary bypass surgery patients were superior to those of fresh liquid-preserved platelets [28]. On the other hand, cryopreserved platelets have also been transfused prophylactically [2,29], and although increments in platelet counts were reported, it is not clear whether the platelets were hemostatically active and safe.

The possible failure of traditional in vitro indicators to truly represent the *in vivo* potential may be the cause of this discrepancy between the results of in vivo and in vitro studies on cryopreserved platelets. In 2013, Dumont et al.’s randomized controlled study provided support for this hypothesis [30]. They evaluated the recovery and survival of 6% dimethyl sulfoxide-frozen autologous platelets in healthy volunteers. They showed that there were no significant differences in functional, morphologic, or in vivo 24-h recovery rates of cryopreserved platelets derived from fresh or 2-day-old irradiated apheresis platelets. They suggested that the accumulating literature knowledge supports proceeding with additional studies to evaluate the clinical effectiveness of cryopreserved platelets [30]. In 2016, Cid et al. revealed that cryopreserved platelets present a phenotype supporting a moderate increase in the rate of clot formation, form stable platelet clots, and do not present a hypercoagulable phenotype during in vitro functional tests [31].

## CONCLUSION

onsidering the damage caused by the freezing process and scarce evidence for in vivo superiority, perhaps frozen platelets should be recommended for austere environments such as combat casualty care, reserving fresh platelets for daily use in blood banks. Therefore, establishment of cryopreserved platelet banks as a part of national contingency plans (natural disasters, large-scale military conflicts, etc.) may be an appropriate strategy. If the utilities of cryopreserved platelets are to be expanded beyond the treatment of combat trauma, such as prophylactic platelet transfusions or regenerative medicine, prospective clinical studies are required to determine their safety and efficacy in well-defined patient cohorts.

## Figures and Tables

**Table 1:**
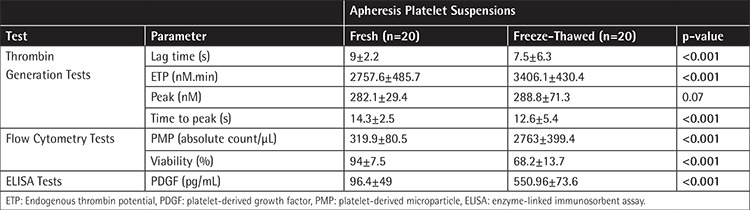
Comparison of the test results of fresh and freeze-thawed apheresis platelet concentrates.
